# Balloon dilation of the eustachian tube: a tympanometric outcomes analysis

**DOI:** 10.1186/s40463-016-0126-6

**Published:** 2016-02-12

**Authors:** Blair Williams, Benjamin A. Taylor, Neil Clifton, Manohar Bance

**Affiliations:** Division of Otolaryngology – Head & Neck Surgery, Department of Surgery, Dalhousie University, Room 3184, 1276 South Park Street, Halifax, NS B3H 2Y9 Canada; Division of Otolaryngology – Head and Neck Surgery, Department of Surgery, St Martha’s Regional Hospital, Halifax, Canada

**Keywords:** Eustachian tube, Eustachian tube balloon dilation, Balloon dilation, Eustachian tube dysfunction, Tuboplasty

## Abstract

**Background:**

Eustachian tube dysfunction (ETD) is a common medical issue, occurring in at least 1 % of the adult population. Patients suffering from ET dysfunction typically present with complaints of hearing loss or sensation of pressure or plugged ear, which can lead to impaired quality of life. Over time ETD can result in conductive hearing loss or choleastatoma formation. Effective theraputic options for ET dysfunction are few. Eustachian tube balloon dilation is a novel surgical technique being used to treat ETD.

The aim of our study is to objectively measure the success of Eustachian tube balloon dilation by comparing pre and post-operative middle ear pressures using tympanometric testing.

**Methods:**

RA retrospective chart review was preformed on all patients who underwent balloon dilation of the Eustachian tube by authors NC or MB from 2010 to 2014. Pre and post-operative tympanograms were analyzed and categorized based on type (Type A, Type B, Type C). Success was defined by an improvement in tympanogram type: Type B or C to Type A, or Type B to type C. Pre and post-operative tympanograms were further analyzed using middle ear pressure values. Follow-up ranged from 3 to 15 months.

**Results:**

Twenty-five ears (18 patients) were included in the study. Overall 36 % of ears had improvement in tympanogram type, and 32 % had normalization of tympanogram post-operatively. The Jerger tympanogram type improved significantly following the procedure (*p* = 0.04). Patients also had statistically significant improvement in measured middle ear pressure post-operatively (*P* = 0.003).

**Conclusion:**

The natural history of Eustachian tube dysfunction is poorly understood, and evidence for current treatments are limited. Eustachian tube balloon dilation is a safe procedure, and produces significant improvement in tympanogram values up to 15 months post-operatively. Further refinement of patient selection and standardization of technique is required to optimize the effect of this therapy. Longterm follow-up data will clarify the persistence of the effect.

## Background

The Eustachian tube (ET) is a conduit between the middle ear space and the nasopharynx, which opens in a physiologically complex and poorly understood manner to provide ventilation to the middle ear, and so equalize middle ear and ambient pressures. In this report, we refer by “Eustachian tube dysfunction” only to dilatory Eustachian tube dysfunction, i.e. failure to open and ventilate the middle ear, as opposed to patulous Eustachian tube, in which there is failure of closure of the Eustachian tube. Eustachian tube dysfunction is a common medical issue, occurring in at least 1 % of the adult population [1]. ET dysfunction can lead to impaired quality of life due to persistent sensation of ear fullness, ear pain, and inability to tolerate air travel or scuba diving. With time, ET dysfunction can lead to conductive hearing loss and cholesteatoma formation.

Patients suffering from ET dysfunction typically present with complaints of hearing loss or sensation of pressure or plugged ear, which can be chronic or recurrent. Findings of ET dysfunction can include serous effusion, conductive hearing loss (on tuning fork or audiometric testing), or negative middle ear pressure (on pneumatic otoscopy or tympanometry). Later, there may be findings of sequelae of this dysfunction, such as retraction pockets, perforations, chronic drainage or cholesteatoma. The underlying etiology and natural history of ET dysfunction is poorly understood. There is a lack of clear diagnostic criteria, which further impairs our ability to study the disease and potential therapies. Anti-reflux therapy or nasal steroid sprays are often used first line treatments, without much evidence to support their efficacy. A randomized, placebo controlled study examining the effect of nasal steroid spray on ET dysfunction found no significant difference between treatment and placebo [2]. Similarly, a recent systematic review found no significant effect of any intervention including observation, nasal steroids, and various surgical techniques [3].

The standard surgical treatment of ET dysfunction is myringotomy and tympanostomy tube placement in the tympanic membrane (TM). This technique allows equalization of middle ear pressure and drainage of fluid via the TM, effectively bypassing the ET. This approach effectively relieves symptoms but does not treat the ET dysfunction. Tympanostomy tubes often need to be replaced multiple times if ET dysfunction persists. This places a burden on the health care system and adds to patient discomfort and inconvenience. Tympanostomy tubes also have some risk of perforations of the tympanic membrane, with associated conductive hearing loss. Other novel surgical therapies have emerged, which focus on the ET itself.

In select patients there is redundant mucosa in the area of the opening of the ET, impairing its dilation. Ablation of this tissue with laser [4] or microdebrider [5] has shown promise in small studies but these interventions are not appropriate for all patients. Other novel therapies have focused on the cartilaginous portion of the ET [6]. Of particular note, a recent, promising innovation is balloon dilation of this portion of the ET.

Eustachian tuboplasty by balloon dilation involves the cannulation of the cartilaginous portion of the ET via the nasopharynx with a balloon catheter. This catheter is inflated to multiple atmospheres of pressure (typically 10–12 bar) for a short amount of time and then removed. The surgical technique is also variable in the literature. Balloons used range between 3–7 mm in diameter, and are of variable lengths. They are typically inflated for 1–2 min. Currently, no evidence exists regarding the optimal balloon diameter, pressure, or duration of inflation.

Numerous studies have demonstrated the safety of this procedure. A systematic review preformed in 2014 showed no adverse outcomes in 103 patients who had undergone balloon dilation of the Eustachian tube [3]. While some short term success has been reported, there is little data regarding long-term outcomes [7–12]. The criteria for measuring surgical success are inconsistent across studies, with outcomes often consisting of subjective symptomatic impressions or non-validated subjective scoring systems. The primary outcome for the present study was middle ear pressure improvement following ET dilation in patients with chronic ET dysfunction. This was accomplished by comparing pre- and post-operative tympanogram values.

## Methods

Approval for this study was obtained from our Nova Scotia Health Authority Research Ethics Board. Data were collected via retrospective chart review. All patients who underwent balloon ET dilation by authors NC or MB, from 2010 to 2014, were reviewed. The procedures were preformed at two different centers but the surgical technique was consistent. The Belfiel® Eustachian tube dilatation system (Spiggle and Theis, Overath, Germany) was used. Under general anesthesia the Eustachian tube orifice was identified endoscopically, and cannulated with a 20 mm long, 3 mm diameter balloon. The balloon was inflated for 2 min at 10 bar and then removed. Surgeon MB also placed tympanostomy tubes in a subset of patients. These patients were not selected, but rather requested concurrent placement of tympanostomy tubes, as this was the standard therapy they were accustomed to. There was no other selection criterion for patients who received a ventilation tube and those who did not.

Patients were selected for ET balloon dilation if they had long-standing Eustachian tube dysfunction (ETD) treated with multiple sets of tympanostomy tubes, and were interested in pursuing a longer-term solution. Patients were excluded from analyses if they had a normal pre-operative tympanogram or an ‘open’ post-operative tympanogram (i.e., a perforation or patent tympanostomy tube). These patients could not be included as a main outcome measure was improvement on the tympanogram, which couldn't be measured for improvement in these cases. If the tympanostomy tube extruded or the perforation healed during the study period, the results were included in analysis. Patients were also excluded if no post-operative tympanograms were performed.

Tympanogram results were collected retrospectively from pre-operative visits and all visits up to 15 months post-operatively. Follow up time points were 2–3 months, 6–9 months, and 12–15 months post operatively. In-hospital audiologists preformed Tympanometric testing. Values were generated using a tympanometer, which produced waveforms and peak pressure values. Tympanograms were then assessed by audiologists (blinded) and again by the attending surgeon (not blinded). Although standard definitions of Type A, B and C tympanograms were used, there is the possibility of interpretation bias. The pre-operative and most recent post-operative tympanograms were categorized based on type (Type A, Type B, Type C) and compared using the Wilcoxon Signed Rank Test. Success was defined by an improvement in tympanogram type: Type B or C to Type A, or Type B to type C.

### Data analysis

Each patient was analyzed by comparing their pre-operative tympanogram and their most recent post-operative tympanogram value. The data was broken down for two different analyses. First, the change in tympanogram Jerger type was analyzed, as this is the most clinically familiar parameter. For this, we counted how many tympanograms evolved from one type (A, B, or C) to another type post-treatment. Data for a second analysis were regrouped to better assess the Type C tympanograms, by analyzing by the actual measured middle ear pressure before and after balloon dilatation. Type C tympanograms were defined as those with the maximum compliance peak at less than -150 dPa. The negative tympanogram values were grouped by 100 daPa intervals. This was assessed first for all patients, (i.e those who did, and did not, receive ventilation tubes in aggregate). Then we repeated the analysis for just the subset that had received ventilation tubes concurrently. This was performed, despite the low numbers, so that we could see if this group had different outcomes compared to the group without concurrent ventilation tubes. Tympanograms for this subset were preformed after the tubes had extruded and the tympanic membrane had healed, not while the tubes were in place. The data were non-parametric, repeated measures so a Wilcoxon Signed rank test was selected to determine statistical significance. Type B tympanograms do not generate a numerical value so our data set could not be analyzed using parametric tests.

## Results

A total of 25 ears were included. Patients ranged in age from 18–68 years, with a mean age of 40.6 years. Follow-up time ranged from 3 to 15 months with a mean follow-up of 7.1 months. All patients had recurrent serous otitis media or negative pressure and retraction, requiring ventilation tube insertions. Nine patients underwent bilateral operations (18 ears), and 6 patients had had previous tympanostomy tube insertion. Previous mastoid surgery or tympanoplasty had been preformed on 4 patients.

**Fig. 1:**
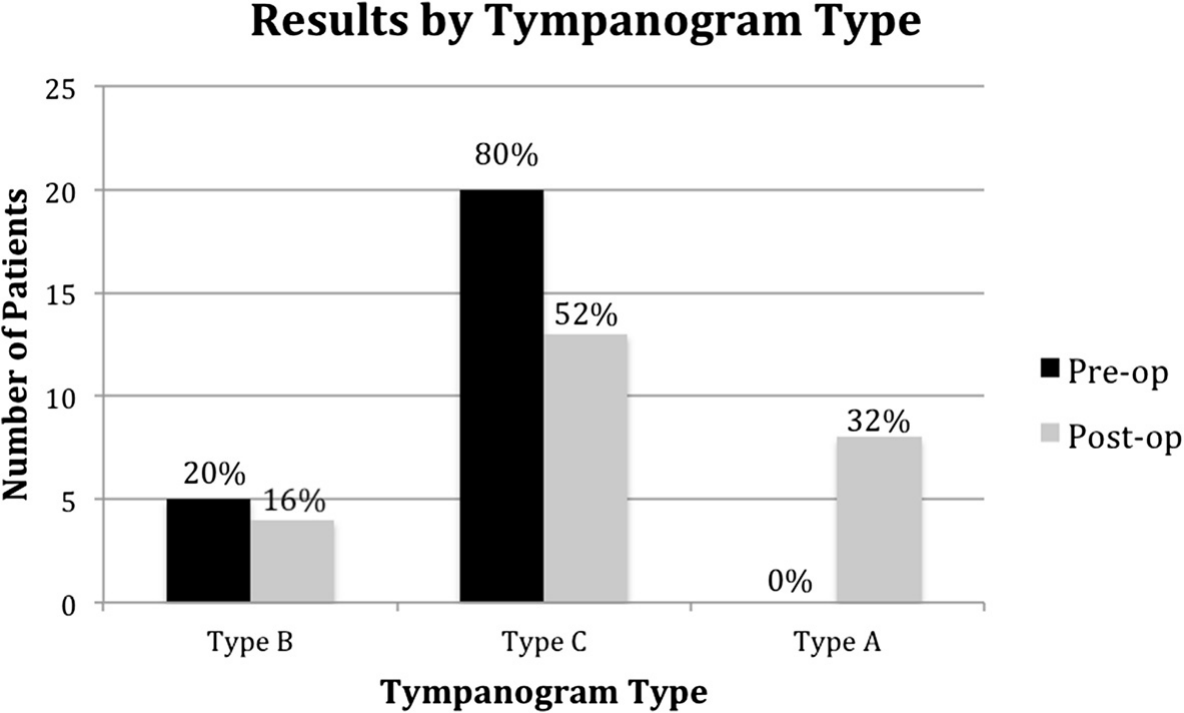
Pre and post-operative assessment of tympanogram type

**Fig. 2:**
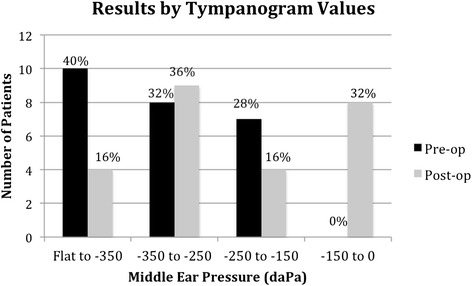
Pre and post-operative grouped tympanogram values

**Fig. 3:**
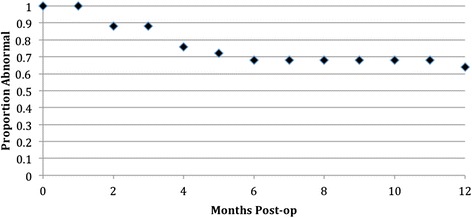
Kaplan Meier survival curve of proportion of abnormal tympanograms at various follow-up points

Tympanograms were preformed at each follow up visit, and the most recent post-operative tympanograms were used for analysis. A summary of the results experienced by individual patients is summarized in Table 1. Overall, 36 % of patients improved their tympanogram type and 32 % had their tympanogram normalize to Type A. Figure 1 shows pre-operative and post-operative results by tympanogram type. The type improved significantly following the procedure (*p* = 0.04). Figure 2 illustrates the results with negative tympanograms (type C) categorized by 100 daPa intervals. Figure 2 appears to show a clearer difference between pre and post operative status. Again, the improvement was significant (*p* = 0.003). At one centre, 5 of 11 ears had concurrent tympanostomy tube placement with ET dilation. In this group, 60 % had normalization of middle ear pressure. In the remaining 6 of 11 ears, 67 % improved in tympanogram type and 50 % normalized. Data on the timing of tympanostomy tube extrusion was not available. Figure 3 illustrates the proportion of abnormal tympanograms pre operatively and at multiple post operative follow up points. The proportion of abnormal tympanograms was highest pre-operatively and decreased most between pre operative and 2–3 month follow up points. All follow up points had a lower proportion of abnormal tympanograms compared to preoperative measures. This was generated through tympanometric analysis at all follow up points. The most negative middle ear pressure pre operatively (*n* = 18, mean = −295 DaPa, SD = 77.38) with the most improvement seen at the 2-3 month follow up point (*n* = 15, mean = −164 DaPa, SD = 105.09). Middle ear pressure at 6–9 months (*n* = 9, mean = −255, SD = 90.08) and at 12-15 months (*n* = 8, mean = −213, SD 124.64) also remained less negative compared to pre-operative state.

**Table 1:** Summary of pre and post-operative improved tympanograms based on type (A, B, or C)

Pre-op tymp	Post-op tymp	Proportion	Percentage
B	A	2/5	40 %
C	A	6/20	30 %
B	C	1/5	20 %
Improvement in Type		9/25	36 %
Normalization of Tympanogram		8/25	32 %

No adverse events occurred as a result of ET balloon dilation.

## Discussion

Eustachian tube balloon dilation has emerged as a surgical option, which targets the cartilaginous portion of the ET. Histopathological analysis preformed after balloon dilation shows decreased inflammation in the surface epithelium and submucosal tissues. The net reduction of inflammation is the hypothesized mechanism for improvement in clinical Eustachian tube function post-operatively [5]. Recent studies have shown promise in both short term and long-term outcomes, but variability in operative approach, sample size, patient follow-up, and outcome measurements make it difficult to interpret with certainty [3, 6–12]. Many studies focus not on tympanometric outcomes, but on ability to valsalva, opening pressures, or subjective outcomes. We feel the most important end-point is whether or not there is return of middle ear ventilation, and have used middle ear pressure as a surrogate measure for this. Our study relied on objective measurements using tympanogram values, and had follow-up to 15 months post operatively.

Overall, 36 % of patients showed an improvement in tympanogram type post-operatively (Table 1) and this improvement was significant (*p* = 0.04, Fig. 1). Similarly, tympanogram measurement categorized by 100 daPa intervals also showed statistically significant improvement post-operatively (*p* = 0.003, Fig. 2). Follow-up time ranged from 3 to 15 months with a mean follow-up of 7.1 months, and the most recent post-operative tympanograms were used for analysis. A recent systematic review of Eustachian tube balloon dilation showed a conversion to type A tympanograms in 36 to 96 % of patients [3]. Silvola et. al reported type A tympanograms in 23 (56 %) patients post operatively, compared to 1 (2 %) pre-operatively, with a similar follow-up time [7]. The lesser benefit in found our study might be due to variability in patient selection, or surgical approach. Silvola et. al reported use of a 7 mm diameter balloon, whereas we used a 3 mm balloon.

Other studies of interest reported outcomes in a summative Eustachian Tube Score (ETS). This score relies on subjective symptoms and tubomanometry to measure successful opening of the Eustachian tube post-operatively with pressurization of the nasopharynx. A higher score indicates improvement in subjective symptoms and lower opening pressures on tubomanometry. For 1076 balloon dilation procedures, Schroder et. al found significant improvement in 71 % of patients at 2 months post-operatively, 73 % at 1 year, and 82 % at 2 years procedures [8]. In a study of 380 cases, Dalchow et. al also showed a mean increase in ETS at 12 months post-operatively [9].

We present the first Canadian data on balloon dilatation of the ET. In our study, the concurrent placement of tympanostomy tubes at the time of ET dilation does not appear to improve outcomes, though sample size of those who received a ventilation tube was small. No literature has shown outcomes of ET balloon dilation with concurrent myringotomy.

Limitations of our study include relatively small sample size, limited longitudinal follow up, and lack of a control group. Due to these limitations, and the lack of a control arm in our study, we cannot say, definitively, if our intervention improved ET function over time, compared to simple observation.

The use of objective outcome measurements is strength of the study, however, we did not collect associated subjective outcomes. Thus, while some middle ear pressures improved, we cannot say if this was related to relief of symptoms or improved quality of life. This consideration would be important for future studies.

Other future directions of this study include the effect of adjunctive interventions (i.e., tympanostomy tube insertion) preformed at the time of Eustachian tube balloon dilation and determining if there are patient factors that correlate with success. Further analysis of surgical protocol and equipment will aid in comparison of results among studies and improve the predictability of patient outcomes. It is important for clinicians to document their results with different diameter balloons, balloon pressures, and dilatation times, so that these parameters can be compared and the surgical approach optimized for success.

## Conclusion

Eustachian tube dysfunction is a common entity that is difficult to treat. Eustachian tube balloon dilation produces modest improvement in tympanogram scores up to 14 months post-operatively. Further refinement of patient selection and standardization of technique is required to optimize the effect of this therapy.

## Abbreviations

CSOM: Chronic serous otitis media
ET: Eustachian tube
ETD: Eustachian tube dysfunction
TM: Tympanic membrane
